# Athero-oncology perspective: identifying hub genes for atherosclerosis diagnosis using machine learning

**DOI:** 10.3389/fimmu.2025.1616096

**Published:** 2025-11-04

**Authors:** Liyan Zhao, Xuzhen Lv, Wen Chen, Xinru Li, Jie Zhou, Qi Ai, Qinhui Tuo

**Affiliations:** ^1^ School of Basic Medicine, Ningxia Medical University, Yinchuan, China; ^2^ Key Laboratory for Quality Evaluation of Bulk Herbs of Hunan Province, School of Pharmacy, Hunan University of Chinese Medicine, Changsha, China; ^3^ Department of Anesthesiology, People's Hospital of Ningxia Hui Autonomous Region, Ningxia Medical University, Yinchuan, China; ^4^ Key Laboratory of Vascular Biology and Translational Medicine, Medical School, Hunan University of Chinese Medicine, Changsha, China

**Keywords:** atherosclerosis, immune infiltration, smooth muscle cells, macrophage, cancer gene, diagnostic biomarker

## Abstract

**Background:**

The transformation of smooth muscle cells (SMCs) into alternative phenotypes is a key process in atherosclerosis pathogenesis. Recent studies have revealed oncological parallels between atherosclerosis and cancer, such as DNA damage and oncogenic pathway activation in SMCs, but the precise molecular mechanisms remain poorly understood. This study integrates cancer gene sets using bioinformatics to identify key hub genes associated with atherosclerosis and explores their immune molecular mechanisms.

**Methods:**

Datasets from the Gene Expression Omnibus (GEO) were analyzed to identify differentially expressed genes (DEGs) and module genes using Limma and WGCNA. Machine learning algorithms (SVM-RFE, LASSO regression, and random forest) were employed to identify cancer-related hub genes for early atherosclerosis diagnosis. A diagnostic model was constructed and validated. UMAP plots from single-cell RNA sequencing data were used to analyze the expression patterns of hub genes, particularly focusing on macrophage-like SMCs in SMC lineage-traced mouse models. Biomarker expression was validated in both human and mouse experiments.

**Results:**

Four cancer-related hub genes (CRGs) were identified: Interferon Regulatory Factor 7 (IRF7), Formin Homology 2 Domain Containing 1 (FHOD1), Tumor Necrosis Factor (TNF), and Zinc Finger SWIM Domain Containing 3 (ZSWIM3). A diagnostic nomogram using IRF7, FHOD1, and TNF demonstrated high accuracy and reliability in both training and validation datasets. Immune microenvironment analysis revealed significant differences between atherosclerosis and control groups. Spearman correlation analysis highlighted associations between hub genes and immune cell infiltration. Single-cell RNA sequencing identified distinct SMC-derived cell clusters and phenotypic transitions, with increased expression of IRF7 and FHOD1 in macrophages potentially derived from SMCs in both human carotid plaques and mouse models.

**Conclusion:**

This study integrates cancer gene sets to identify key hub genes in atherosclerosis, emphasizing its parallels with cancer. The diagnostic nomogram based on IRF7, FHOD1, and TNF provides a reliable tool for early diagnosis, while insights into SMC phenotypic switching and immune microenvironment modulation offer potential therapeutic targets.

## 1 Introduction

Atherosclerosis (AS) is a leading cause of morbidity and mortality worldwide, contributing significantly to the global burden of cardiovascular diseases (CVD) (1–3). It is characterized by arterial narrowing and thrombotic events triggered by unstable plaque rupture or erosion, leading to severe outcomes such as myocardial infarction or stroke. The progression of AS is driven by complex genetic and cellular mechanisms, emphasizing the need for ongoing research to identify key therapeutic targets and biomarkers for early diagnosis and intervention.

A concept that has recently emerged to understand the molecular mechanisms of AS is “athero-oncology.” (4). This term refers to the parallels between atherosclerosis and cancer, drawing attention to shared biological processes that contribute to the development of both diseases. In the context of AS, “athero-oncology” highlights the role of cancer-related pathways in AS pathogenesis, particularly the processes of inflammation, cellular proliferation, and metabolic reprogramming. These pathways, commonly associated with cancer progression, also drive the progression of atherosclerosis, making this concept an important framework for exploring shared molecular mechanisms.

Chronic inflammation is a hallmark of both cancer and atherosclerosis. In cancer, inflammation plays a pivotal role in tumor initiation, progression, and metastasis. Similarly, in AS, inflammatory processes are critical in driving plaque formation, with immune cells infiltrating the arterial walls and producing cytokines that exacerbate the disease (5, 6). Furthermore, proliferation is another key mechanism shared between cancer and atherosclerosis. In cancer, uncontrolled cell division drives tumor growth, whereas in AS, the clonal proliferation of smooth muscle cells (SMCs) contributes to plaque buildup and vascular remodeling (7, 8). Additionally, metabolic reprogramming, a hallmark of cancer cells, is also implicated in atherosclerosis. In both diseases, alterations in lipid metabolism, including the dysregulation of bioactive lipids like S1P, PGE2, and LPA, promote cellular proliferation and inflammation, thereby driving disease progression (9, 10).

The molecular mechanisms linking AS and cancer are not yet fully understood. However, DNA damage, a hallmark of cancer, has been observed in SMCs within atherosclerotic lesions. Recent research has shown that these SMCs display tumor-like characteristics, including genomic instability, excessive proliferation, and activation of oncogenic pathways (11, 12). For example, the expression of the KrasG12D oncogene in SMCs has been found to accelerate the progression of AS, while the anticancer drug niraparib has demonstrated potential therapeutic effects in AS mouse models (4). These findings have led to the emergence of “athero-oncology” as a framework for studying the shared mechanisms between AS and cancer, offering insights into both basic and translational research.

Given the critical role of SMCs in AS pathogenesis, the advent of high-throughput genomics has enabled the identification of differentially expressed genes (DEGs) involved in this process. Combining bioinformatics and machine learning, we analyzed microarray and single-cell RNA-sequencing datasets from the GEO database to investigate the involvement of cancer-related genes (CRGs) in AS. Additionally, we explored the association between CRGs and immune infiltration to provide insights into the immunometabolic interplay underlying AS progression.

## 2 Materials and methods

### 2.1 Data collection

**Figure 1:**
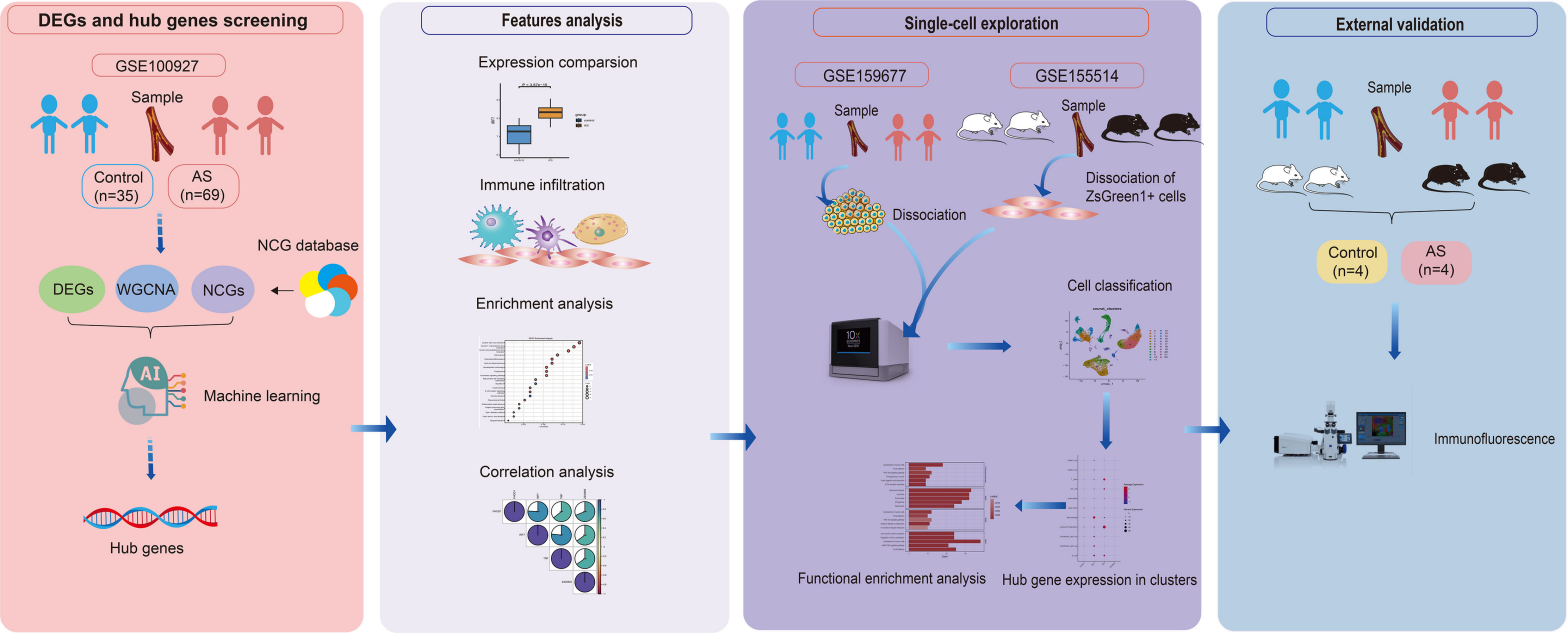
Flow chart of the study.

The data analysis methods for this study are detailed in **Figure 1**. Original datasets from GEO series GSE100927, GSE43292, GSE159677, GSE28829and GSE155514 were obtained from the NCBI Gene Expression Omnibus database (http://www.ncbi.nlm.nih.gov/geo), an essential resource for genomic data. Detailed descriptive information of datasets was shown in **Table 1**.

**Table 1:** Descriptive statistics of the GEO datasets.

GEO accession	Platform	Experiment type	Sample		Species
			Control	AS	
GSE100927	GPL17077	array	35	69	Homo sapiens
GSE28829	GPL570	array	13	16	Homo sapiens
GSE43292	GPL6244	array	32	32	Homo sapiens
GSE159677	GPL18573	single-cell RNA-seq	3	3	Homo sapiens
GSE155514	GPL24247	single-cell RNA-seq	1	3	Mus musculus

### 2.2 Identification of DEGs

Data from the GEO database were systematically retrieved and analyzed using the “GEOquery” package (version 2.70.0), designed specifically for managing GEO datasets (13). Differential expression gene (DEG) analysis utilized the “limma” package (version 3.58.1), a commonly used method for gene expression analysis (14). Visualization tools such as “ggplot2” (version 3.5.0) for volcano plots and “ pheatmap” (version 1.0.12) for heatmaps were employed to enhance the intuitive interpretation of the data.

### 2.3 Functional enrichment analysis

Gene set enrichment analysis (GSEA) was conducted using two approaches: KEGG pathway analysis via the gseKEGG function from the clusterProfiler package(version 4.10.0) and MSigDB Hallmark Pathways analysis using the same package’s GSEA function (15). Both methods utilized gene identifiers converted for respective databases. Enrichment significance was determined using a p-value threshold of <0.05, allowing for a focused exploration of biological pathways implicated in our data. Comprehensive functional enrichment analyses were carried out to explore the potential functions of identified targets. This involved Gene Ontology (GO) analysis, which categorized genes by molecular functions (MF), biological pathways (BP), and cellular components (CC). Furthermore, a Kyoto Encyclopedia of Genes and Genomes (KEGG) enrichment analysis was conducted to correlate gene functions with extensive genomic data, thereby deepening the understanding of the roles of AS-related target genes. These analyses utilized the “clusterProfiler” and “GOplot” packages (version 1.0.2), renowned for their effectiveness in functional analysis.

### 2.4 Weighted gene coexpression network analysis

To dissect complex gene interactions and identify modules of correlated genes, this study applied Weighted Gene Co-expression Network Analysis (WGCNA). This method provides a systematic approach to studying the correlation patterns among genes and their relationship to phenotypic traits, offering potential insights into underlying biological processes (16).

The initial phase of the analysis focused on pre-processing the gene expression data to normalize the values and exclude statistical outliers, ensuring the integrity of the network analysis. The selection of the soft-thresholding power was critical and was determined based on the criterion of achieving a scale-free topology fit index close to 0.9, thus optimizing the network for both sensitivity and robustness. The minModuleSize parameter was set to 30 to ensure that modules with fewer than 30 genes were excluded from the analysis. The mergeCutHeight was set to 0.25, indicating that modules with a dissimilarity (1- correlation) greater than 0.25 were merged. The deepSplit parameter was kept at the default value, which did not significantly alter the module formation.

Following the establishment of a suitable threshold, genes were clustered using an unsupervised hierarchical clustering approach. The dynamic branch cutting method was then employed to define modules, each represented by a distinct color for straightforward identification and analysis. For each module, calculations of module membership (MM) and gene significance (GS) scores were conducted. MM evaluated the correlation of individual genes with the overall module, whereas GS assessed the correlation of genes with specific external traits being studied. Modules showing high MM and substantial GS scores were selected for further investigation. The study focused on hub genes within these modules, identified due to their significant intramodular connectivity. These genes are hypothesized to play pivotal roles in gene regulatory networks and were therefore subjected to further analysis to elucidate their functional contributions to the phenotype in question.

### 2.5 Machine learning-based identification of central hub genes

Advanced machine learning techniques were utilized to identify critical hub genes in AS. The process began with the application of the support vector machine (SVM) algorithm, a robust supervised learning method, for modeling using a select set of feature genes. This technique is advantageous for managing high-dimensional data by focusing on maximizing the margin between different classes. Following this, SVM-recursive feature elimination (SVM-RFE) was applied to iteratively refine the feature set, removing the least significant features to boost the model’s predictive accuracy, essential for isolating the most informative genes for AS diagnosis. Further refinement was achieved using Least Absolute Shrinkage and Selection Operator (LASSO) regression via the “glmnet” package (version 4.1.8). LASSO is celebrated for its capability in variable selection and regularization, aiding in the prevention of model overfitting. The selection within the LASSO model adhered to the 1-SE criterion, maintaining a balance between model complexity and performance. For cross-validation, 10-fold cross-validation was applied to assess model stability and avoid overfitting. The random forest algorithm was then used to rank genes based on their importance. Random forest is an ensemble learning method, and in this study, we used 500 trees to enhance the stability and robustness of the model. This method is particularly effective for managing unbalanced data and estimating feature importance, which is crucial for pinpointing key genes. Genes with a relative importance score exceeding 0.25 were deemed significant. The final selection of hub genes was based on an intersection analysis of the outcomes from LASSO logistic regression, SVM-RFE, and random forest methods, ensuring a thorough and robust selection process.

### 2.6 Development of nomograms and evaluation via ROC curves

A nomogram incorporating hub genes was developed using the “rms” package (version 6.7.1) to enhance the diagnostic accuracy of AS. Each gene in the nomogram was assigned specific points based on its contribution to AS diagnosis, with the total points aggregating the scores of all genes. This graphical tool simplifies complex genetic data, providing clinicians with an intuitive method to assess patient risk. Diagnostic efficacy was evaluated using Receiver Operating Characteristic (ROC) curves and Precision-Recall (PR) curves. The area under the curve (AUC) and its 95% confidence intervals (CIs) were calculated using the “pROC” package (version 1.18.5), employing a nonparametric approach suitable for various datasets. The DeLong method, used for AUC calculation, assesses the model’s ability to discriminate between AS and control groups, where values closer to 1 indicate higher accuracy. The Precision-Recall (PR) curve was used to evaluate the model’s performance, particularly in datasets with class imbalance, with the PR-AUC providing insight into the model’s predictive power for identifying the positive class.

External validation was performed using the GSE43292 and GSE28829 datasets, ensuring the model’s robustness and applicability across different cohorts. Additionally, Decision Curve Analysis (DCA) was employed to assess the clinical utility of the nomogram, comparing its net benefit across different threshold probabilities.

### 2.7 Single-sample gene set enrichment analysis methodology

The study utilized the “Gene Set Variation Analysis” (GSVA) package in R to conduct single-sample gene set enrichment analysis (ssGSEA), assessing pathway variations and biological processes in individual samples (17). This approach highlighted the diversity of immune responses in AS patients by measuring the infiltration of 28 immune cell types and pinpointing gene set differences between AS and control groups. For data comparison, nonparametric tests were applied. The Wilcoxon rank-sum test was used to compare outcomes between groups without presuming a normal distribution of the data. Additionally, Spearman correlation analysis was conducted to explore the relationships between immune cells, enhancing the understanding of their interactions in AS.

To address potential concerns regarding algorithm selection and to validate the robustness of our immune infiltration findings, we performed a complementary analysis using the CIBERSORT algorithm. CIBERSORT employs a linear support vector regression approach to deconvolve the expression matrix and estimate the relative proportions of 22 immune cell types based on the LM22 signature matrix. We compared these results with those obtained from our primary ssGSEA analysis to assess the consistency and concordance between the two widely used methods.

### 2.8 Single-Cell RNA-sequencing data analysis

In our research, we analyzed single-cell transcriptome data from two datasets, GSE155514 and GSE159677, both sourced from the GEO database. Dataset GSE159677 includes samples from calcified atherosclerotic core (AC) plaques and corresponding proximal adjacent (PA) sections of the carotid artery, obtained from three patients undergoing carotid endarterectomy. Conversely, dataset GSE155514 encompasses samples from atherosclerotic plaques and associated vessels in mice, offering a comparative animal model perspective. The dataset GSE155514 was derived from a SMC-lineage tracing murine model developed by crossing *ROSA26* ^ZsGreen1/+^ mice with *Myh11-CreER* ^T2^mice. In this model, after tamoxifen induction, SMCs and their progeny permanently expressed the fluorescent protein ZsGreen1, facilitating the identification of these cells *in vivo*. ZsGreen1 expression showed high concordance with the SMC marker ACTA2 in normal sections of the brachiocephalic artery. To explore SMC dynamics during atherogenesis, *ROSA26* ^ZsGreen1/+^; *Myh11-CreER* ^T2^ mice were bred onto an *Ldlr* ^−/−^ background, enhancing our ability to trace SMC behavior under atherosclerotic conditions. Analysis was conducted using single-cell RNA sequencing. Data preprocessing was performed with the Seurat R package (version 5.0.1), renowned for its efficacy in single-cell genomic analysis, to ensure the accuracy and reliability of the results. Essential metrics such as the number of molecules per cell (nCount RNA) and the number of genes detected per cell (nFeature RNA) were evaluated alongside sequencing read counts to verify data integrity. Mitochondrial genomic contamination, indicative of low-quality or dead cells, was assessed by calculating the percentage of reads mapping to the mitochondrial genome, utilizing the percentage feature set function in Seurat. Cell clustering involved using the dimensionality reduction method of unified manifold approximation and projection (UMAP) after filtering principal components, facilitating clear visual identification of cell clusters. Cell marker genes with statistically significant adjusted p-values (<0.05) were identified and utilized to categorize the clustered cells.

### 2.9 Human atherosclerotic samples

To validate the expression of the critical biomarker in human atherosclerotic samples, carotid atherosclerotic plaques were obtained from patients undergoing carotid endarterectomy due to carotid artery stenosis. The plaques were designated as the model group, while the adjacent vascular endothelium within 0.5 cm of the plaque margin was used as the control group. Each group included four samples. The study was conducted at the People’s Hospital of Ningxia Hui Autonomous Region, with approval from the hospital’s Ethics Committee. Written informed consent was obtained from all participants prior to enrollment.

### 2.10 Mice atherosclerotic samples

For the animal experiments, 10 male ApoE^-/-^mice, aged 6 weeks, were obtained from Beijing HFK Bioscience Co.,Ltd. (Beijing, China). Prior to the experiment, all mice were adaptively fed for 2 weeks. To induce atherosclerosis, five mice were transitioned from a normal chow diet (NCD) to a high-fat diet (HFD) at 8 weeks of age. The HFD consisted of 77.5% standard chow, 20% lard, 2% cholesterol, and 0.5% sodium cholate. These mice were fed the HFD for 12 weeks. The remaining five mice continued on the NCD for 12 weeks and served as the control group. At the end of the study, all mice were anesthetized by intraperitoneal injection of pentobarbital sodium (60 mg/kg) and subsequently euthanized for the collection of aortic tissue samples. All animal experimental protocols were reviewed and approved by the Ethics Committee of Hunan University of Chinese Medicine (Hunan, China).

### 2.11 Immunofluorescence staining

Paraffin-embedded tissue sections of human and mouse arterial tissues were prepared for immunofluorescence staining. The sections were deparaffinized, rehydrated, and subjected to antigen retrieval before staining. Samples were incubated overnight at 4°C with primary antibodies, including IRF7 (1:50; Cat# 22392-1-AP; Proteintech) and FHOD1 (1:50; Cat# PC13158s; Abmart). Primary antibody binding was detected using a CoraLite^®^488-conjugated goat anti-rabbit IgG secondary antibody (1:1000; Cat# ab150081; Abcam). For colocalization studies, sections were costained with a CD68 primary antibody (1:50; Cat# E-AB-22013; Elabscience), followed by detection with an Alexa Fluor^®^ 594-conjugated goat anti-mouse IgG secondary antibody (1:1000; Cat# ab150120; Abcam).Nuclei were counterstained with Fluoroshield containing 4′, 6-diamidino-2-phenylindole (DAPI; Cat# F6057; Sigma, USA). Fluorescent images were captured using the Nikon A1R HD25 confocal microscope system (Nikon, Japan). Image processing and fluorescence quantification were performed using Fiji (ImageJ).

### 2.12 Statistical analyses

The human and mouse immunofluorescence data are presented as mean ± standard deviation. Comparisons were made using an unpaired t-test, with a two-tailed P-value < 0.05 considered statistically significant. All statistical analyses and graph generation were conducted using GraphPad Prism V8.4.3 software.

## 3 Results

### 3.1 Identification of DEGs between atherosclerotic lesions and control arteries

**Figure 2:**
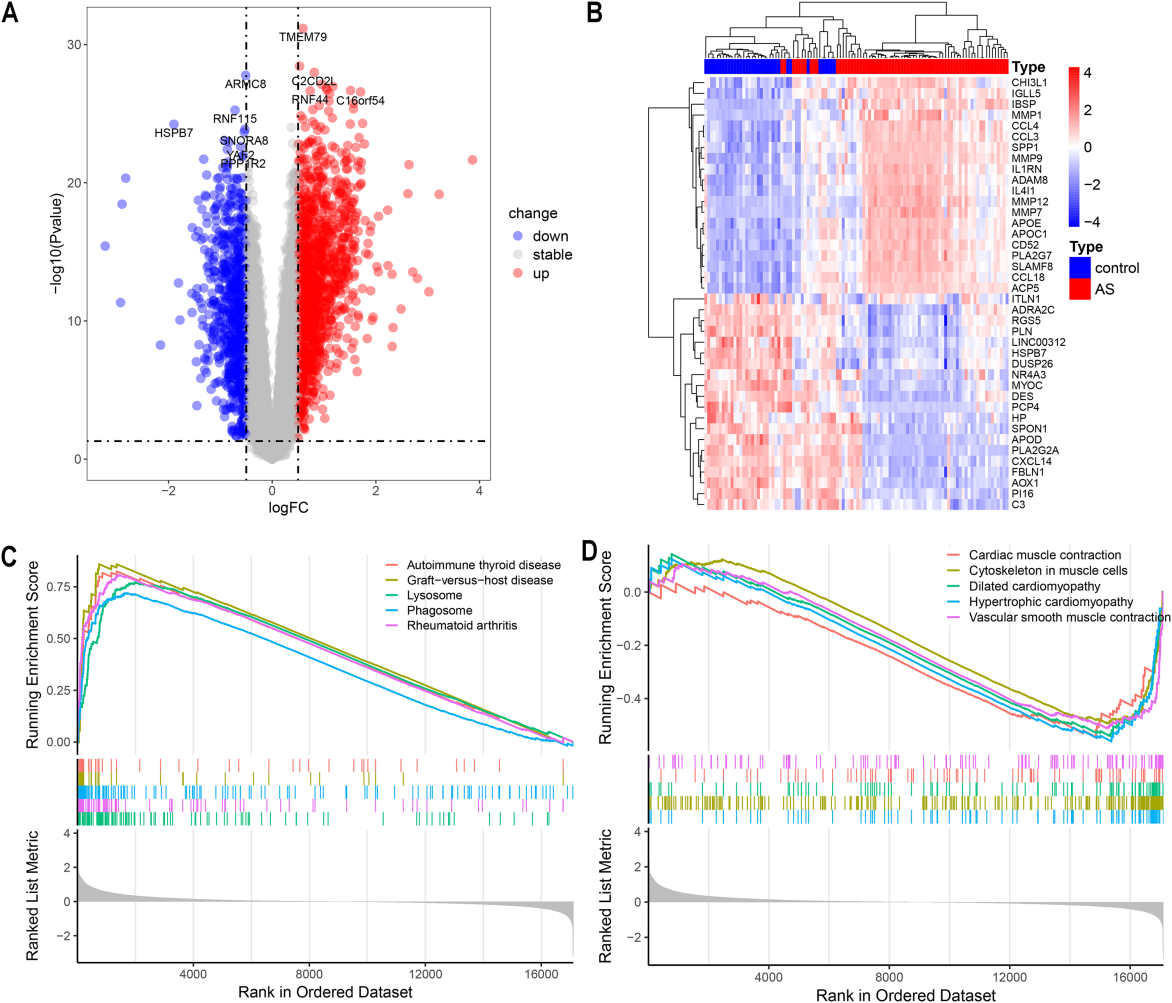
Analysis of Differentially Expressed Genes (DEGs). **(A)** Volcano plot showing up/down-regulated DEGs of the AS vs. control groups in the GSE100927 dataset. **(B)** Heatmap depicting DEGs expression patterns in the GSE100927 dataset. **(C, D)** Gene set enrichment analysis (GSEA) reveals the principal upregulated and downregulated pathways from the KEGG database in the GSE100927 dataset.

The study’s flowchart is depicted in **Figure 1**. *P*-values were adjusted using the Benjamini-Hochberg’s false discovery rate (FDR) method, and genes with an adjusted *P*-value < 0.05 and |log2 fold-change (log2FC)| > 0.5 were considered differentially expressed. A total of 2318 differentially expressed genes (DEGs) were identified in the GSE100927 dataset, including 907 downregulated and 1411 upregulated genes, as summarized in **Supplementary Table S1**. The volcano plot in **Figure 2A** and the heatmap in **Figure 2B** effectively demonstrate the differences in gene expression between atherosclerotic lesions (AS group) and control arteries (control group), with the heatmap highlighting the top 20 upregulated and downregulated genes. The evaluation of pathway enrichment was performed by comparing the pathways between the AS and control groups using GSEA. In the AS group, the allograft rejection, graft-versus-host disease, lysosome, phagosome and rheumatoid arthritis were significantly enriched (**Figure 2C**). Conversely, cardiac muscle contraction; cytoskeleton in muscle cells; dilated cardiomyopathy; hypertrophic cardiomyopathy and vascular smooth muscle contraction were significantly downregulated (**Figure 2D**). For these top pathways identified in the GSEA analysis, Normalized Enrichment Scores (NES) and FDR q-values were provided, with a q-value cutoff of < 0.05. Detailed results are presented in **Supplementary Table S2**. These pathways reveal that in AS, the activity of lysosomes and phagosomes, alterations in the cytoskeleton of muscle cells, and the abnormal contraction of vascular smooth muscle cells collectively trigger inflammatory responses, promote structural remodeling of the vascular wall, and ultimately drive plaque formation.

### 3.2 Weighted gene coexpression network construction

**Figure 3:**
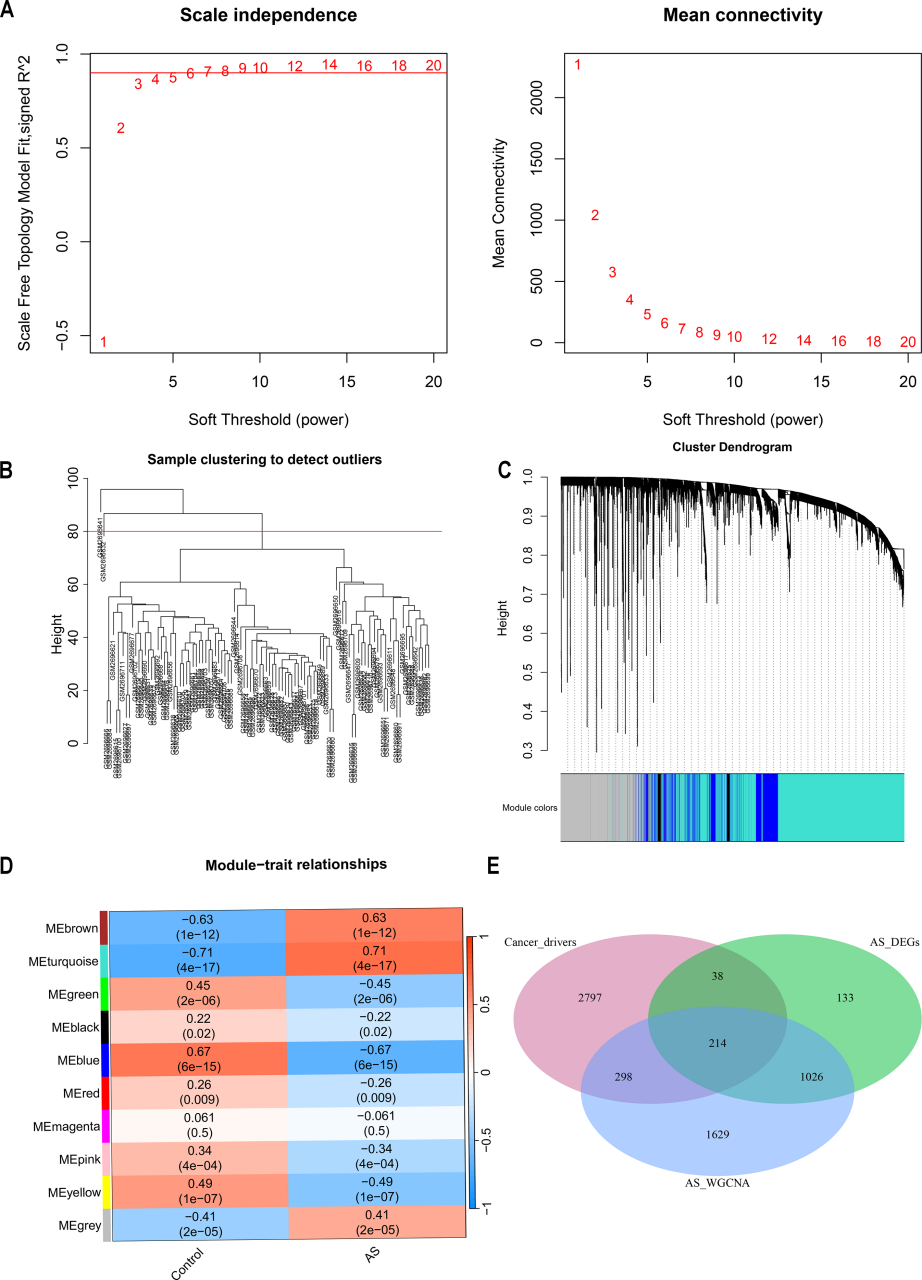
Enrichment levels in the weighted gene coexpression network analysis (WGCNA) of the genome. **(A)** Soft threshold β = 14 and scale-free topological fit index ­(R^2^). **(B)** Each leaf represents a unique sample, and the red line indicates the height threshold for outlier detection. **(C)** the hierarchical clustering of gene modules. **(D)** Heat map of module-trait correlations. **(E)** Venn Diagram of cancer drivers, AS differentially expressed genes (DEGs), versus genes identified in AS WGCNA.

The GSE100927 dataset, sourced from the GEO data repository, consisted initially of 69 atherosclerotic lesions and 35 normal samples. After discarding two low-quality atherosclerotic lesions, 67 atherosclerotic lesions alongside the normal samples underwent clustering as depicted in **Figure 3B**. A soft thresholding power of 14, applied when R^2^ exceeded 0.9 and average connectivity was high, is detailed in **Figure 3A**. This process identified 10 distinct modules for deeper analysis. The relationship between these modules and clinical symptoms was explored through frontal correlations between module eigengene (ME) values and clinical features, with the turquoise and brown modules showing the strongest positive correlations with both control and AS groups, as illustrated in **Figure 3D**. The two significant modules identified were considered clinically relevant, and all the genes contained within these modules were selected for further investigation, as detailed in **Supplementary Table S3**.

### 3.3 Identification and functional analysis of CRGs in the context of AS

Cancer genes were obtained from the NCG 7.1 database, which includes 591 canonical cancer drivers and 2756 candidate cancer drivers, totaling 3347 genes. Utilizing the WGCNA method, key module genes positively correlated with AS trait expression were identified and analyzed alongside upregulated DEGs in AS derived from the limma method. This analysis pinpointed 1240 overlapping genes, from which 214 were recognized as cancer-related genes (CRGs), overlapping with cancer driver genes (**Figure 3E**, **Supplementary Table S4**). Further functional analysis was conducted on these CRGs. The GO enrichment analysis delineates the pivotal roles in regulating cell differentiation, modulating immune responses, and orchestrating the complex intracellular processes of substance uptake, transport, and processing (**Supplementary Figure S1A**). KEGG pathway analysis links these CRGs to essential processes such as immune responses, lipid metabolism, and cell differentiation, underscoring their pivotal roles in the progression of atherosclerosis (**Supplementary Figures S1B, C**).

### 3.4 Identification of key hub genes through machine learning techniques

**Figure 4:**
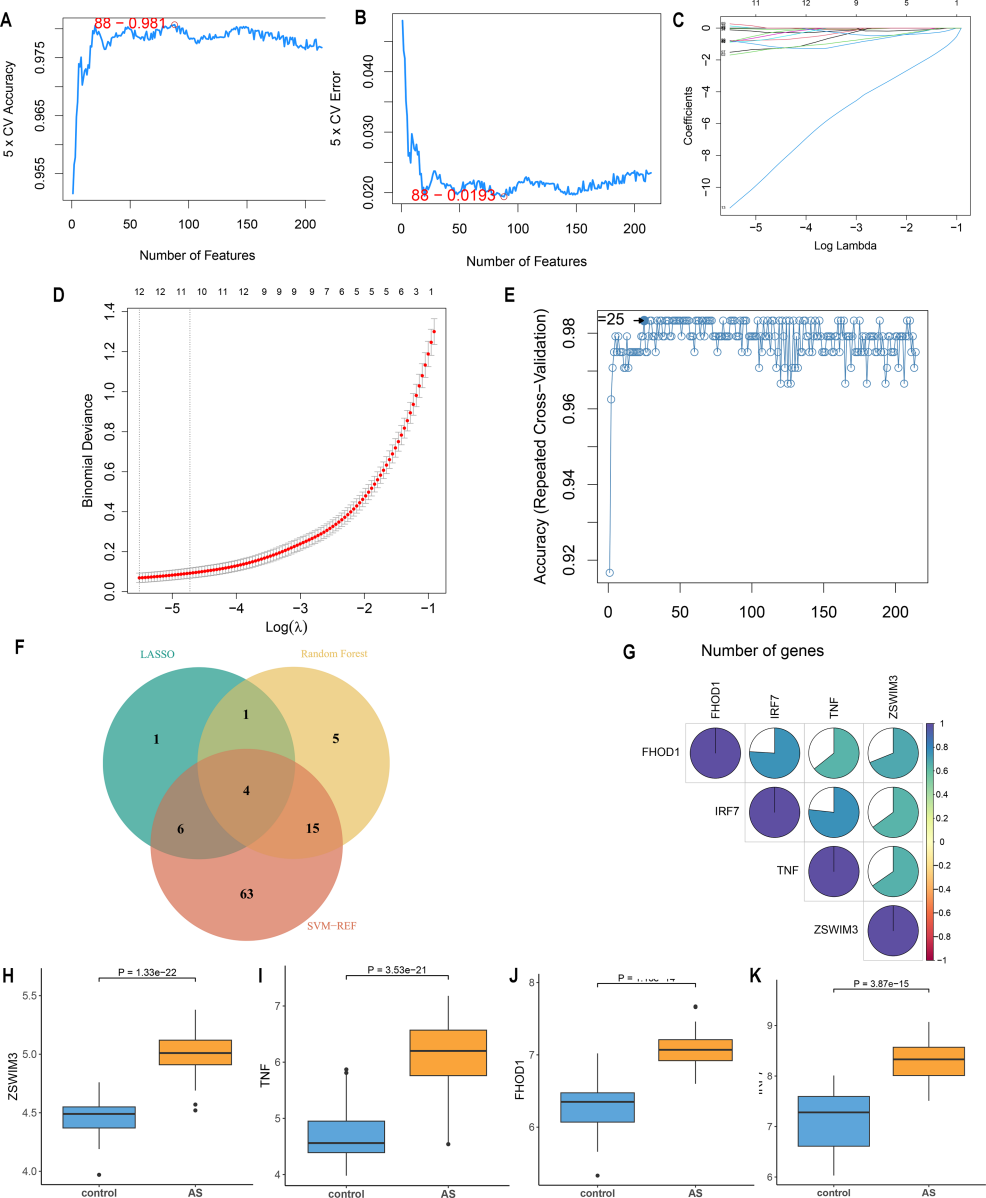
Feature gene selection. **(A, B)** Signature gene expression was screened based on the support vector machine recursive feature elimination (SVM-RFE) algorithm. **(C, D)** Adjusting feature selection using the least absolute shrinkage and selection operator (LASSO) algorithm. **(E)** Random forest error rate versus the number of classified trees. **(F)** Venn diagram of the four hub genes obtained from the intersection of results from SVM-RFE, RF, and LASSO algorithms **(G)** Correlation between hub genes. **(H-K)** Expression of four hub genes in AS and control groups.

Three machine-learning algorithms were used to screen feature genes among the set of 214 CRGs. Specifically, SVM-RFE identified 88 genes with the highest accuracy of 0.981 and the lowest error of 0.019 (**Figures 4A, B**; **Supplementary Table S5**); LASSO regression analysis predicted 12 genes among the statistically significant univariate variables (**Figures 4C, D**; **Supplementary Table S6**); random forest and feature selection were employed to determine the relationship between error rate, classification tree numbers, and 25 genes with relative importance (**Figure 4E**; **Supplementary Table S7**). To obtain a robust gene signature for AS, genes that overlapped among the three aforementioned methods were obtained using a Venn diagram. Four hub genes, namely FHOD1, IRF7, TNF and ZSWIM3 were obtained as shown in **Figure 4F**. The correlation analysis depicted in **Figure 4G** shows that FHOD1, IRF7, and TNF have positive correlations with each other, as indicated by the blue sections in the circular plots. FHOD1, IRF7, TNF, and ZSWIM3 showed a significant increase in the AS group compared to the controls, as illustrated in **Figures 4H–K**.

The nomogram model developed in this study was trained using plaque tissue samples, making it a crucial “tissue-based research classifier” for diagnosing atherosclerosis. To assess its clinical translational potential, we plan to validate its diagnostic signature using RNA from blood samples, PBMCs, or extracellular vesicles in future research. This additional validation will help determine its applicability for non-invasive clinical testing, enhancing its translational relevance for future clinical use.

### 3.5 Modeling and testing a diagnostic nomogram model for AS

**Figure 5:**
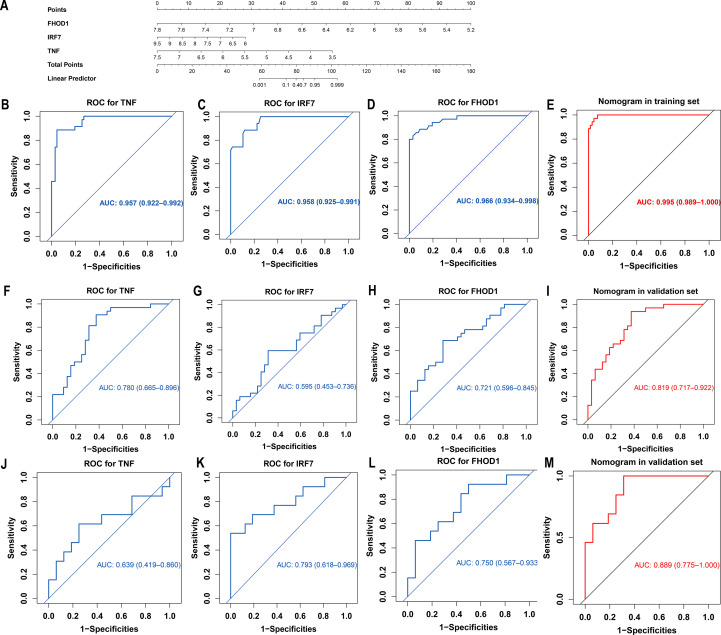
Development and validation of a diagnostic nomogram for atherosclerosis based on hub genes identified by machine learning. **(A)** Nomogram constructed using the GSE100927 training dataset, incorporating three hub genes (TNF, IRF7, FHOD1) for atherosclerosis diagnosis. Points are assigned for each gene expression level and summed to calculate the total points and corresponding disease probability. **(B-E)** ROC curves for each hub gene (TNF, IRF7, and FHOD1), as well as the combined nomogram, demonstrating their significant diagnostic value in the GSE100927 training set. **(F-I)** ROC curves for each hub gene (TNF, IRF7, and FHOD1), as well as the combined nomogram, demonstrating their significant diagnostic value in the GSE43292 validation set. **(J-M)** ROC curves for each hub gene (TNF, IRF7, and FHOD1), as well as the combined nomogram, demonstrating their significant diagnostic value in the GSE28829 validation set.

Due to significant multicollinearity between ZSWIM3 and other variables, model fitting was unsuccessful, leading to the exclusion of ZSWIM3 from the model fitting process. A diagnostic nomogram incorporating three key genes—TNF, IRF7, and FHOD1—was developed (**Figure 5A**). The model’s performance was first evaluated using ROC curves, with AUC values and 95% confidence intervals (CIs) calculated for each gene in the GSE100927 training set (**Figures 5B–D**). The AUC values for TNF (AUC = 0.957, 95% CI: 0.922–0.992), IRF7 (AUC = 0.958, 95% CI: 0.925–0.991) and FHOD1 (AUC = 0.966, 95% CI: 0.934–0.998), indicated excellent performance in the training set.

The nomogram demonstrated outstanding diagnostic accuracy in the GSE100927 training set with an AUC of 0.995 (95% CI: 0.989–1.000), suggesting a near-perfect model fit (**Figure 5E**). The AUC values for the nomogram in the GSE43292 and GSE28829 validation datasets were 0.819 (95% CI: 0.717–0.922) and 0.889 (95% CI: 0.775–1.000), respectively (**Figures 5I, M**), further confirming the nomogram’s robustness across independent datasets.

For the GSE43292 validation set, the AUC values for the individual genes were as follows: TNF (AUC = 0.780, 95% CI: 0.665–0.896), IRF7 (AUC = 0.595, 95% CI: 0.453–0.736), and FHOD1 (AUC = 0.721, 95% CI: 0.596–0.845) (**Figures 5F–H**). In the GSE28829 validation set, the AUC values for TNF (AUC = 0.639, 95% CI: 0.419–0.860), IRF7 (AUC = 0.793, 95% CI: 0.618–0.969), and FHOD1 (AUC = 0.750, 95% CI: 0.567–0.933) were also promising (**Figures 5J–L**).

The Precision-Recall (PR) curve for the training set showed a PR-AUC of 0.991, reflecting excellent performance. For the validation sets, the PR-AUCs were 0.802 (GSE43292) and 0.871 (GSE28829), indicating solid but slightly reduced performance in external datasets (**Supplementary Figures S2A, D, G**). The calibration curve demonstrated excellent agreement between predicted probabilities and observed outcomes across all datasets. In the training set (GSE100927), the calibration slope was 0.90, with a negligible intercept of 0.09, indicating minimal overestimation and high accuracy (mean absolute error = 0.021) (**Supplementary Figure S2B**). In the validation sets, the calibration slopes were 0.95 (GSE43292) and 0.86 (GSE28829), with corresponding intercepts of 0.04 and 0.10, showing good calibration and acceptable accuracy (mean absolute errors of 0.049 and 0.083, respectively) (**Supplementary Figure S2E, H**). Decision Curve Analysis (DCA) demonstrated strong clinical utility for the nomogram. In all datasets, the model outperformed “all” and “none” strategies, indicating its robust ability to identify high-risk AS patients (**Supplementary Figures S2C, F, I**). In summary, the nomogram showed robust diagnostic performance across training and validation datasets, with high AUC, strong PR-AUC, and favorable DCA, confirming its reliability and clinical utility for AS diagnosis.

### 3.6 Enrichment analysis of the hub genes

An in-depth investigation into the biological functions of the four central genes—FHOD1, IRF7, TNF, and ZSWIM3—was conducted in the AS group. Using GSEA, we explored the Hallmark Pathways in mSigDB, focusing on the differential expression between high and low levels of these genes. The results (**Supplementary Figures S3A–D**) revealed significant enrichment in pathways such as TNFα signaling via NF-κB, inflammatory response, interferon-gamma response, allograft rejection, KRAS signaling, and IL6-JAK-STAT3 signaling. These pathways are canonical drivers in cancer, where they contribute to tumor progression, immune evasion, and metastasis. Notably, these same pathways are involved in the progression of AS, with dysregulation potentially contributing to SMC phenotypic switching and plaque formation.

Further analysis using ssGSEA highlighted biological differences between AS and control groups. The correlation between signature gene expression and ssGSEA scores, analyzed using the “corrplot” package (**Supplementary Figure S3E**), revealed strong associations with hallmark gene sets, including apoptosis, coagulation, complement, IL2-STAT5 signaling, interferon-gamma response, interferon-alpha response, and IL6-JAK-STAT3 signaling. These findings suggest that the dysregulation of cancer-related pathways, such as KRAS and IL6-JAK-STAT3, plays a critical role not only in tumor microenvironment remodeling but also in the pathological processes of AS, such as SMC transdifferentiation and plaque progression.

### 3.7 Immune cell infiltration in AS

Current experimental and clinical research supports the role of immune mechanisms in hastening the progression of AS (18). In both various cancers and atherosclerotic plaques, elevated levels of inflammatory molecules not only promote cell proliferation by providing growth signals but also facilitate cell transdifferentiation (19, 20). This encourages the investigation of the relationship between key signatures and immune infiltration in AS. To ensure the robustness of our immune infiltration assessment, we compared the results from our primary ssGSEA analysis with those generated by the CIBERSORT algorithm. We observed a strong positive correlation between the two methods for the majority of immune cell types (**Supplementary Figure S4**), particularly for macrophages. This high concordance reinforces the validity of our findings regarding the differential immune infiltration patterns between AS and control groups. While both methods yielded consistent results, we opted to present ssGSEA as our primary approach due to its versatility and fewer data requirements. Specifically, ssGSEA does not rely on cell-type specific gene signatures, making it applicable to a broader range of datasets, including those with limited immune cell gene signature data. Moreover, ssGSEA is less dependent on reference datasets, which allows it to be more adaptable and reproducible across different study contexts, making it a more suitable method for our analysis of immune infiltration in atherosclerosis.

**Figure 6:**
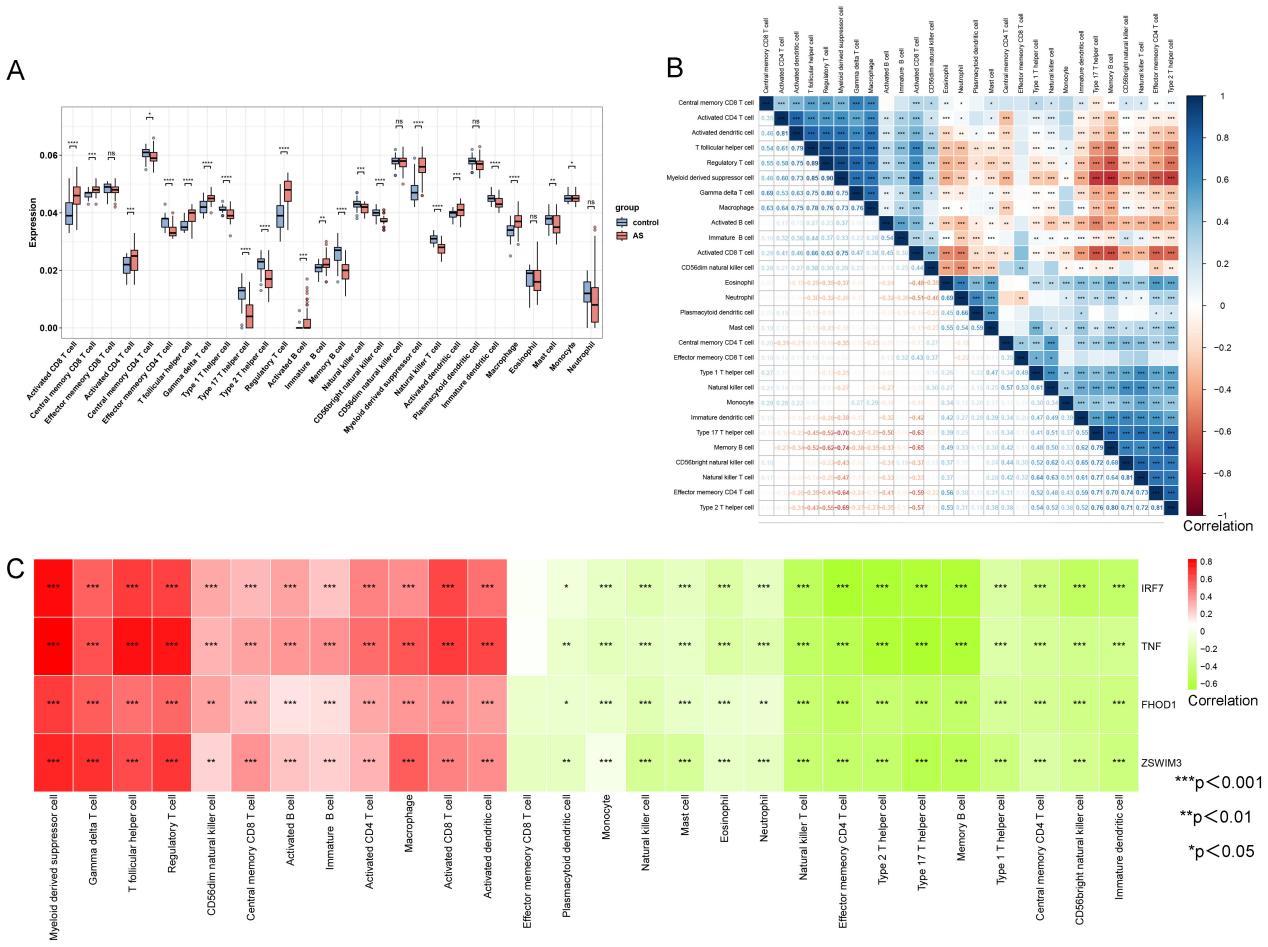
Comparison of immune cell type infiltration between AS and controls (GSE100927 dataset) assessed by ssGSEA. **(A)** Boxplot illustrating the proportions of immune cells. **(B)** Correlation matrix of immune cell proportions. *p < 0.05, **p < 0.01, ***p < 0.001. **(C)** Correlation analysis between hub genes and immune cells.

The ssGSEA algorithm assessed the infiltration of 28 immune cell types in the AS and control groups from the GSE100927 dataset to explore variations in their immune profiles. Significant differences in the infiltration of various immune cells were noted within atherosclerotic plaques. Of the 28 immune cell types analyzed, only five—memory CD8 T cells, CD56dim natural killer cells, plasmacytoid dendritic cells, eosinophils, and neutrophils—did not display significant differences (P < 0.05) between the AS and control groups, as depicted in **Figure 6A**. Further investigation, as shown in **Figure 6B**, highlighted notable correlations among these cells, quantified by specific scores. The analysis revealed strong synergistic interactions between memory activated CD4 T cell and activated dendritic cell (0.81), regulatory T cell and T follicular helper cell (0.89), myeloid-derived suppressor cell and regulatory T cell (0.90), natural killer T cell and CD56 bright natural killer cell (0.81), and type 2 T helper cell and effector memory CD4 T cell (0.81). The correlation analysis between the four upregulated hub genes (IRF7, TNF, FHOD1, and ZSWIM3) and immune cell infiltration in AS showed a predominantly relationship (**Figure 6C**). Specifically, the upregulated expression of these genes was strongly correlated with increased infiltration of immune cells, including Macrophages, Gamma delta T cells, T follicular helper cells, and several others. This suggests that these hub genes in AS may be associated with the modulation of immune cell activity, potentially contributing to the progression of AS through immune regulation.

### 3.8 Single−cell RNA−seq analysis

We utilized scRNA-seq to analysis the expression level and location of four hub genes. The data are sourced from the dataset GSE159677. Cells with gene detection counts per cell exceeding 5000 or falling below 200, as well as cells with mitochondrial percentages exceeding 5%, were excluded to ensure data quality. UMAP revealed the presence of 11 distinct cell clusters, each labeled with a distinct color (**Supplementary Figure S5A**). Considering the expression patterns of marker genes, the clustering results obtained through UMAP were further refined and annotated using single R and Cell-Marker (**Supplementary Figure S5A**). Compared to the control group, there was a reduction in contractile smooth muscle cells (VSMCs-a), an increase in synthetic smooth muscle cells (VSMCs-b), a decrease in endothelial cells, and an increase in immune cells (**Supplementary Figure S5B**). The expression pattern of four hub genes was depicted in the UMAP plots. In the AS group, there was an increase in the number of macrophages compared to the control group, accompanied by a significant upregulation of IRF7 and FHOD1 in these cells (**Supplementary Figures S5C, D**). TNF is primarily expressed in T cells, while ZSWIM3 is expressed at low levels across all cell types (**Supplementary Figures S5C, D**). Foam cell formation is a hallmark of the early phase of AS. Growing evidence has demonstrated that most foam cells in AS lesions are formed from VSMCs that have transdifferentiated into macrophages and subsequently taken up lipids (21, 22). However, it remains unclear whether IRF7 and FHOD1 are upregulated in foam cells derived from VSMCs. Therefore, we analyzed single-cell transcriptome data (GSE155514) from atherosclerotic plaques and vessels in mice to further investigate whether IRF7 and FHOD1 are upregulated in these VSMC-derived macrophages.

### 3.9 Integrating single-cell genomics with SMC-lineage tracing to uncover diverse SMC-derived cell states in AS

**Figure 7:**
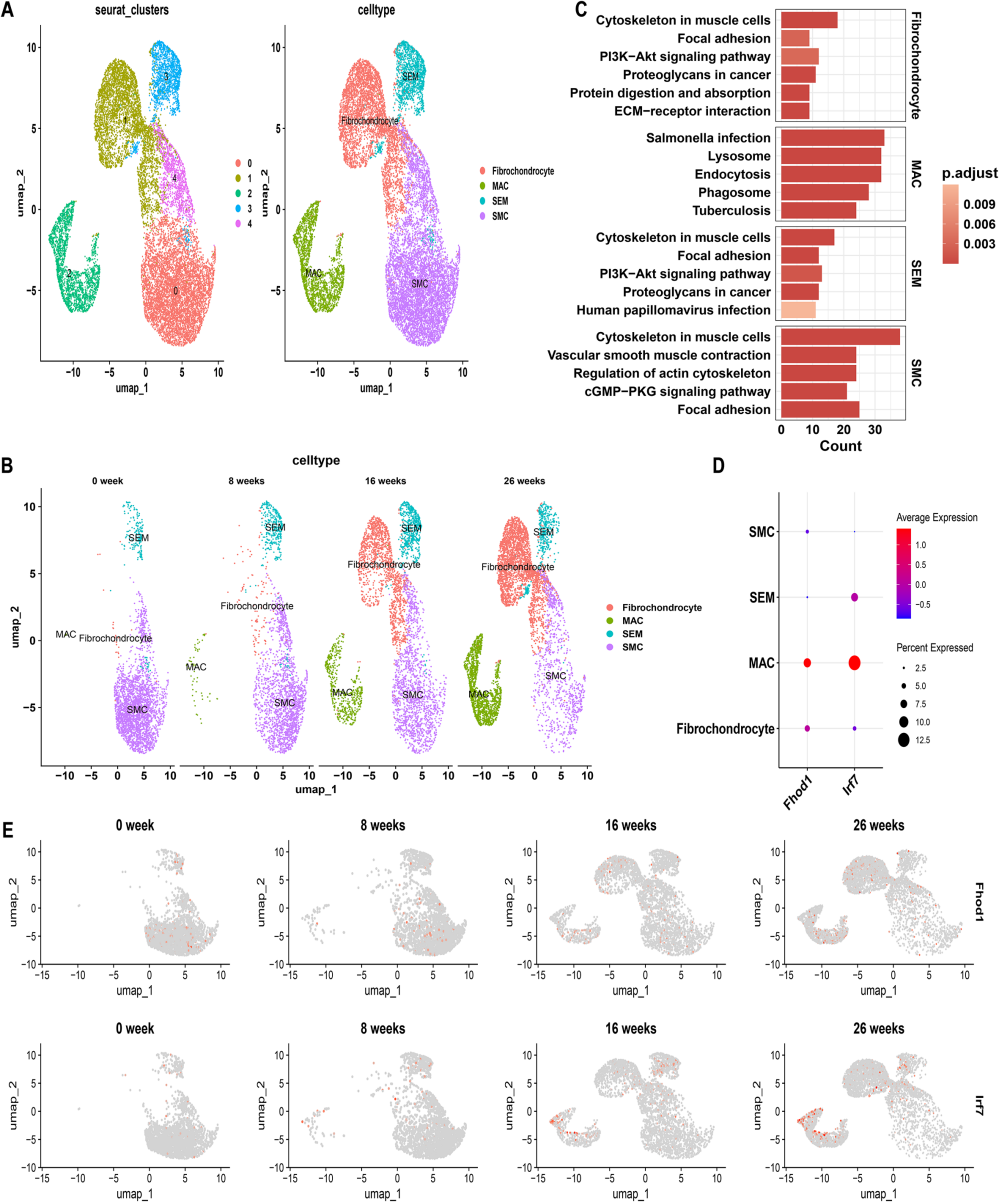
Single-cell genomics with SMC-Lineage Tracing: Normalized Data Comparison. **(A)** Unified manifold approximation and projection (UMAP) clustering into 5 clusters, and Cells were annotated using CellMarker and singleR. **(B)** UMAP visualization illustrating the dynamic changes in cellular populations over a 26-week period. **(C)** KEGG pathway enrichment in different VSMC subtypes. **(D)** Feature Plots showing the expression pattern of FHOD1 and IRF7 over a 26-week period. **(E)** Dot plot shows the expression levels of FHOD1 and IRF7 in each cell cluster.

The single-cell transcriptome data from GSE155514 were obtained from a SMC-lineage tracing murine model developed by crossing *ROSA26* ^ZsGreen1/+^ mice with *Myh11-CreER* ^T2^mice. Cells with gene detection counts per cell exceeding 4000 or falling below 200, as well as cells with mitochondrial percentages exceeding 5%, were excluded to ensure data quality. UMAP revealed the presence of 5 distinct cell clusters, each labeled with a distinct color (**Figure 7A**). Cell clusters based on ZsGreen1^+^ status at all time points (0, 8, 16, 26 weeks) indicated that multiple SMC-derived cell types and states emerged over time during AS. These include the original contractile SMCs, SMC-derived ICS, an intermediate cell state later termed “SEM” cells, fibrochondrocytes, and macrophage-like cells (MACs) (**Figure 7B**).

To discern phenotypic disparities among VSMC subtypes, we performed KEGG analysis on DEGs identified in each cell type, revealing cell-type-specific activated signaling pathways (**Figure 7C**). This result was also in consistent with characteristic gene profiles, defining 4 VSMC subtypes by functional annotation: SMC represented the original, biological type of VSMC for enriched vascular smooth muscle contractile function and genes. Fibrochondrocytes were enriched in ECM-receptor interaction. SEM, an intermediate cell state, demonstrated both SMC and fibroblast characteristics, with synthetic genes as indicated by molecular traits. MACs displayed a proinflammatory signature, characterized by phagosome involvement and inflammation-related signaling pathways (**Figure 7C**). UMAP analysis at different time points revealed that with the progression of AS, the number of contractile SMCs gradually decreased, while SMC-derived SEM cells, fibrochondrocytes, and MACs increased (**Figure 7B**). These findings highlight the plasticity of SMCs and their critical contribution to the cellular heterogeneity observed in AS lesions. Interestingly, as the proportion of MACs increased, the expression levels of IRF7 and FHOD1 were also upregulated in these cells over time (**Figures 7D, E**). In contrast, both IRF7 and FHOD1 exhibited minimal expression in contractile SMCs at week 0, suggesting that these genes are not actively expressed in SMCs under homeostatic conditions. However, with the progression of AS, and as SMCs transitioned into MAC-like cells, the expression of IRF7 and FHOD1 became markedly increased in the MAC population.

### 3.10 Expression of IRF7 and FHOD1 in atherosclerotic patients and mice

**Figure 8:**
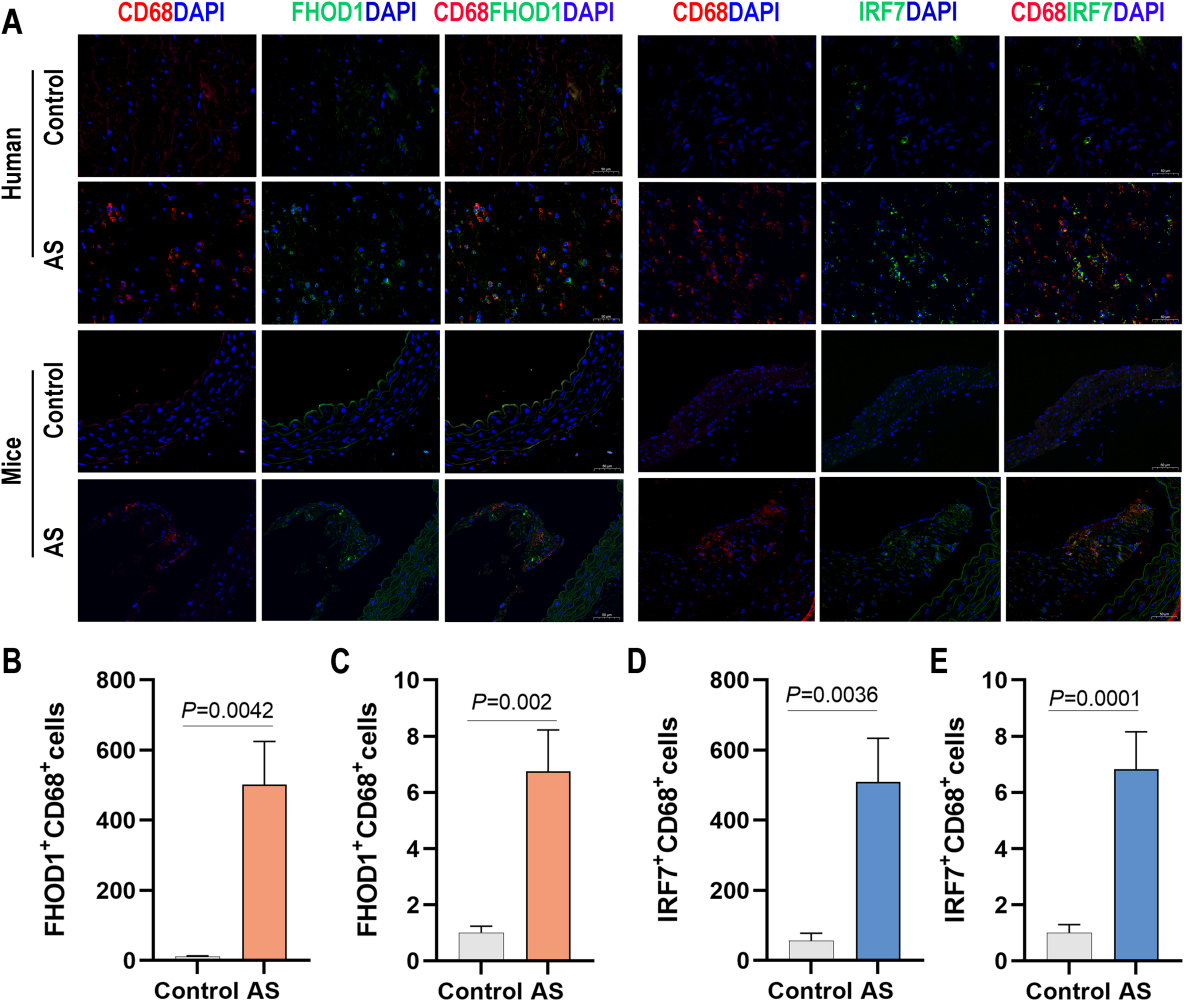
Expression of FHOD1 and IRF7 in human and mouse atherosclerotic tissues. **(A)** Immunofluorescence staining of FHOD1 (left) and IRF7 (right) with the macrophage marker CD68 in human atherosclerotic tissues, including carotid atherosclerotic plaques and the proximal vascular tissue of plaques, and in aortic arch tissues from WD-fed ApoE^–/–^ mice. **(B, C)** Quantification of the percentage of FHOD1-positive macrophages (CD68^+^ cells) in human **(B)** and mouse **(C)** tissues. **(D, E)** Quantification of the percentage of IRF7-positive macrophages (CD68+ cells) in human **(D)** and mouse **(E)** tissues. Data are presented as mean ± SD (n = 4 per group). Statistical analysis was performed using paired two-tailed Student’s t-test for human tissues and unpaired two-tailed Student’s t-test for mouse tissues.

To further investigate the role of IRF7 and FHOD1 in atherosclerosis, we measured their expression in both human and mouse atherosclerotic tissues. Immunostaining analysis revealed that IRF7 and FHOD1 were colocalized with CD68-positive macrophages in both human and mouse atherosclerotic plaques (**Figure 8**). In the atherosclerotic plaques of mice, we observed that the expression of IRF7 and FHOD1 colocalized with CD68-positive macrophages was significantly increased in the model group compared to the control group. Similarly, in human atherosclerotic plaques and adjacent vascular tissues, elevated expression of IRF7 and FHOD1 colocalized with CD68-positive macrophages was also observed. Taken together, these findings suggest that IRF7 and FHOD1, as potential diagnostic markers, are specifically localized in macrophages within atherosclerotic lesions.

## 4 Discussion

AS is a chronic inflammatory disorder characterized by interactions among lipid-driven processes, immune responses, and vascular remodeling. Traditional approaches to identifying causative genes in AS often overlook the parallels between cancer biology and plaque progression. In this study, we leveraged the concept of “athero-oncology” by integrating oncogene datasets into our bioinformatics analysis of AS-related genes, aiming to uncover novel molecular targets and diagnostic tools for the disease.

First, by performing differential gene expression analysis on public GEO datasets, we identified a broad pool of candidate genes implicated in AS. These genes were then refined using WGCNA, which allowed us to pinpoint modules highly correlated with disease traits. By intersecting this set of genes with established oncogenes, we isolated 214 common genes with potential oncogenic and atherogenic roles. This “hybrid” filtering step is particularly noteworthy, as it illuminates how aberrant phenotypes in AS may mirror tumor-like behaviors such as excessive proliferation, evasion of regulatory checkpoints, and heightened metabolic demands. Moreover, the identified genes are closely linked to immune responses, cell differentiation, and lipid metabolism, reinforcing the proposition that AS is significantly driven by immune and inflammatory pathways, in alignment with contemporary perspectives (23).

To further refine these 214 genes, we employed complementary machine-learning algorithms—random forest, SVM-RFE, and LASSO—each possessing distinct strengths in feature selection. Through this ensemble-based approach, we narrowed down the gene set and arrived at three key genes—IRF7, FHOD1, and TNF—that not only display robust diagnostic potential but also may function as central regulators of plaque development. The subsequent construction of a nomogram model, validated in a separate dataset, underscores the diagnostic utility of these three genes.

Numerous innate immune cells, including macrophages, dendritic cells (DCs), monocytes, mast cells, and neutrophils, are critically involved in the progression of AS (24, 25). In our study, we identified that DEGs in AS samples were primarily enriched in immune regulatory pathways, accompanied by a notable increase in activated immune cells compared to normal controls. Recognizing the pivotal role of immunity in AS, we aimed to explore the relationship between specific gene signatures and immune cell interactions. Our analysis revealed that the genes FHOD1, IRF7, and TNF exhibited variable correlations with distinct immune cells, including macrophages, B cells, and T cells. By integrating single-cell RNA sequencing data, we identified that FHOD1 and IRF7 are predominantly expressed in macrophages within atherosclerotic lesions. In atherosclerotic mouse models with single-cell genomics and SMC lineage tracing, the expression of FHOD1 and IRF7 was significantly upregulated in macrophages derived from SMCs as the disease progressed, while their expression was minimal in homeostatic SMCs. This suggests a close link between the upregulation of these genes and the phenotypic transition of SMCs into MACs. To further validate these findings, we examined the expression of FHOD1 and IRF7 in macrophages within human atherosclerotic plaques and murine atherosclerotic models. Immunofluorescence staining of both human and murine plaques revealed significant co-localization of FHOD1 and IRF7 with CD68^+^ macrophages. Notably, the expression levels of FHOD1 and IRF7 were substantially higher in atherosclerotic plaques compared to controls. These findings suggest that FHOD1 and IRF7 are predominantly expressed in macrophages that may originate from SMCs undergoing phenotypic switching in AS. The phenotypic transition of SMCs into MACs likely plays a critical role in the functional reprogramming of SMCs, contributing to the inflammatory and remodeling processes characteristic of advanced atherosclerotic lesions. The upregulation of FHOD1 and IRF7 in MACs suggests their involvement in mediating these pathological processes. Further investigation is warranted to elucidate the precise molecular roles of FHOD1 and IRF7 in SMC phenotypic modulation and their contribution to the progression of AS. Understanding how these genes regulate SMC-to-MAC transitions may reveal new mechanisms of SMC plasticity and vascular inflammation, highlighting FHOD1 and IRF7 as potential therapeutic targets to mitigate vascular remodeling and atherosclerosis progression.

As the primary vascular cell type, SMCs are crucial for providing mechanical support and facilitating vasoactive responses that maintain vascular homeostasis (26). Dysfunction in these cells can lead to various vasculopathies. However, most current treatments for AS focus on lowering low-density lipoprotein cholesterol (LDL-C) but have minimal direct effects on SMCs. Directly targeting SMCs offers a promising therapeutic approach, particularly for patients with coronary artery disease (CAD) who maintain normal cholesterol levels or experience recurrent CAD despite lipid-lowering therapy.

In the realm of vascular biology, while contractile SMCs provide a stable cellular basis, remodeled SMCs demonstrate reduced intrinsic properties and changes in behavior such as proliferation, migration, and differentiation (27). A key factor in AS progression is the phenotypic switching of specific SMCs within the arterial wall. These cells undergo proliferation, migration, and transdifferentiation, which impacts the stability of lesions and influences the clinical outcomes of the disease (28). Studies using human genetics, single-cell profiling, and SMC lineage tracing reveal that SMCs and their SDCs predominate in AS. While certain SDC subtypes may influence disease outcomes positively or negatively, the precise roles of SMCs and SDCs in AS progression and related clinical complications remain unclear.

Research indicates that the phenotypic transformation of SMCs during AS closely resembles tumor biology, characterized by genomic instability, tumor-like traits, activation of oncogenic pathways, and sensitivity to therapies targeting DNA damage repair (4). These findings, along with insights into clonal hematopoiesis in atherogenesis, have led to the proposal of ‘athero-oncology’ as a framework to unify research in CVD related to AS (4). FHOD1 belongs to the formin family, a group of evolutionarily conserved actin nucleating proteins present in all eukaryotic cells (29). The function of formins is regulated by Rho GTPases, molecular switches that modify the cytoskeleton across various cellular contexts. FHOD1 plays a critical role in cancer progression, enhancing epithelial-mesenchymal transition (EMT), cell migration, and ECM degradation (30). It is notably upregulated in cancers such as oral squamous cell carcinoma and basal-like breast cancer, facilitating EMT-related transformations and increased tumor aggressiveness, especially in triple-negative breast cancer where it influences cytoskeletal dynamics (31, 32). Furthermore, FHOD1’s overexpression in glioblastoma and melanoma underscores its role in promoting tumor invasion and metastasis, marking it as a potential target for therapeutic interventions and a marker of advanced disease stages in cancers like gastric cancer (33, 34). However, despite its established significance in cancer biology, the role of FHOD1 in AS remains poorly understood. To date, only one *in vitro* study has implicated FHOD1 in regulating SMC phenotypes, and no research has explored its involvement in the transformation of SMCs into foam cells or its potential contribution to atherogenesis.

IRF7, a multifunctional transcription factor, regulates cell differentiation, proliferation, and apoptosis, and also participates in immune regulation. Similar to the role of FHOD1 in cancer progression, IRF7 is critical in the development and metastasis of tumors (35). Research on IRF7 shows its diverse roles in cancer. Overexpression of IRF7 boosts IFN-b production and NK cell activity, reducing prostate cancer metastasis (36). It also suppresses survival and invasiveness in gastric cancer (37). However, miR-762 downregulates IRF7, enhancing breast cancer proliferation and invasion (38). Similarly, miR-1587 promotes M2 polarization of macrophages, aiding breast cancer progression (39). Additionally, IRF7 inhibits granulocytic suppressor cells, decreasing lung cancer metastasis (40). However, no studies have yet reported on the role of IRF7 in the transformation of SMCs into foam cells during the process of AS. The formation of foam cells derived from VSMCs is the result of multiple factors acting together, including inflammatory responses (41), lipid metabolism disorders (42), and oxidative stress (43, 44). Based on these studies, we hypothesize that FHOD1 and IRF7 may be involved in lipid-driven inflammatory responses, which are increasingly considered a key pathogenic mechanism in AS. Therefore, our research findings have a certain level of rationality.

The innovation of our approach lies in merging oncogenic signatures with AS datasets to expose potentially critical genes overlooked by traditional AS-focused analyses. The resulting identification of FHOD1 and IRF7—two genes previously recognized for their roles in cancer pathogenesis—points to shared molecular pathways between oncogenesis and advanced atherogenesis. This perspective could foster new therapeutic directions, such as repurposing oncological interventions to moderate proliferative and inflammatory aspects of AS. For instance, the PARP inhibitor niraparib, an approved anti-cancer drug, has been shown to attenuate AS in mouse models (4). This is highly plausible based on the shared mechanism of genomic instability and DNA damage response between cancer and the phenotypic switching of SMCs, a process our study and others have linked to the activation of oncogenic pathways. The efficacy of niraparib in AS demonstrates that by targeting this common vulnerability, this class of drugs can inhibit the aberrant proliferation and survival of SMC-derived cells within atherosclerotic plaques, thereby stabilizing lesions and mitigating disease progression. This example powerfully demonstrates and concretely illustrates the translational potential of the “athero-oncology” framework.

Nevertheless, several limitations warrant attention. First, although we verified pivotal findings in mouse models, extending these observations to human samples is essential to establish clinical relevance. Human validation is particularly critical for the broader application of our findings. To enhance the specificity of the study, it may be beneficial to consider spatial transcriptomics or multiple ion beam imaging (MIBI) as possible next steps. These advanced techniques could allow for the simultaneous resolution of the spatial localization and cell-type-specific markers of IRF7 and FHOD1 expression in human plaques, thus bridging the gap between bioinformatics analyses and *in vivo* validation. Second, our study relied on publicly available data without patient-level clinical information, limiting our capacity for prognostic analysis. Finally, in this study, we identified key hub genes, such as FHOD1 and IRF7, and found a significant correlation between these genes and SMC-derived macrophages in AS progression. While these genes are associated with SMC phenotypic switching and immune modulation, their exact roles remain unclear and need further functional and mechanistic validation. Follow-up *in vivo* and *in vitro* studies are required to confirm the roles of FHOD1, IRF7, and related targets in AS development. As AS is a multifactorial disease, dissecting how these genes regulate foam-cell formation, lesion progression, and plaque stability in the context of both lipid and immune dysregulation will be pivotal for translating our discoveries into actionable therapeutic strategies.

## 5 Conclusions

In conclusion, our integrative bioinformatics pipeline, enriched with oncological insights, offers a novel perspective to understand AS pathogenesis. By identifying and validating the association of hub genes, such as FHOD1 and IRF7, with SMC-derived macrophages, we provide a new conceptual framework that parallels oncogenic processes in atherosclerosis. However, it is important to emphasize that the relationships observed in this study are correlational, and future studies should focus on functional validation to clarify the exact roles of these genes in the progression of AS. We anticipate that this “athero-oncology” approach will stimulate further research into targeted interventions, ranging from early diagnostics to novel therapeutic strategies, to reduce the burden of AS.

## Data Availability

The public datasets utilized in this study were obtained from the GEO database with the following accession numbers: GSE100927, GSE43292, GSE159677, and GSE155514. Cancer-related genes were retrieved from the NCG 7.1 database. The source codes for all bioinformatics analyses are available in the GitHub repository: https://github.com/zhaoliyan5158/cancer-and-AS.

## Supplementary material

Supplementary Figure 1 Functional analysis of cancer-related genes (CRGs) in AS. **(A)** GO analysis. **(B, C)** KEGG analysis.

Supplementary Figure 2 Performance evaluation of the machine learning-based atherosclerosis diagnostic prediction model across independent validation datasets. **(A–C)** Results from the training dataset GSE100927: **(A)** Precision-Recall curve (PR-AUC = 0.991). **(B)** Calibration curve (slope = 0.90, intercept = 0.09). **(C)** Decision curve analysis comparing the model with “All” and “None” strategies. **(D–F)** Validation results from dataset GSE43292: **(D)** Precision-Recall curve (PR-AUC = 0.802). **(E)** Calibration curve (slope = 0.95, intercept = 0.04). **(F)** Decision curve analysis. **(G–I)** Validation results from dataset GSE28829: **(G)** Precision-Recall curve (PR-AUC = 0.871). **(H)** Calibration curve (slope = 0.86, intercept = 0.10). **I** Decision curve analysis. All calibration curves were generated using bootstrapping with 1000 repetitions.

Supplementary Figure 3 Relationship between hallmark pathways and hub genes. **(A-D)** GSEA analysis of the hub genes, highlighting the top 6 enriched pathways for **(A)** TNF, **(B)** IRF7, **(C)** FHOD1, and **(D)** ZSWIM3. **(E)** Correlation analysis between the hub genes and hallmark pathways.

Supplementary Figure 4 Comparison of immune cell type infiltration between AS and controls (GSE100927 dataset) assessed by CIBERSORT. **(A)** Boxplot illustrating the proportions of immune cells. **(B)** Correlation matrix of immune cell proportions. *p < 0.05, **p < 0.01, ***p < 0.001. **(C)** Correlation analysis between hub genes and immune cells.

Supplementary Figure 5 Single-Cell analysis after normalization in the GSE159677 dataset. **(A)** Unified manifold approximation and projection clustering into 22 clusters, and Cells were annotated using CellMarker and singleR. **(B)** UMAP Visualization: The plot distinctly represents cellular populations in both AS and controls. **(C)** Feature Plots showing the expression pattern of FHOD1, IRF7, TNF, and ZSWIM3 in calcified atherosclerotic plaque from AS and control groups. **(D)** Dot plot shows the expression levels of hub genes in each cell cluster.
