# Supplementary figures and images for: Athero-oncology perspective: identifying hub genes for atherosclerosis diagnosis using machine learning
Source: Front Immunol. 2025 Nov 4;16:1616096. doi: 10.3389/fimmu.2025.1616096 (PMC12623321; doi:10.3389/fimmu.2025.1616096)

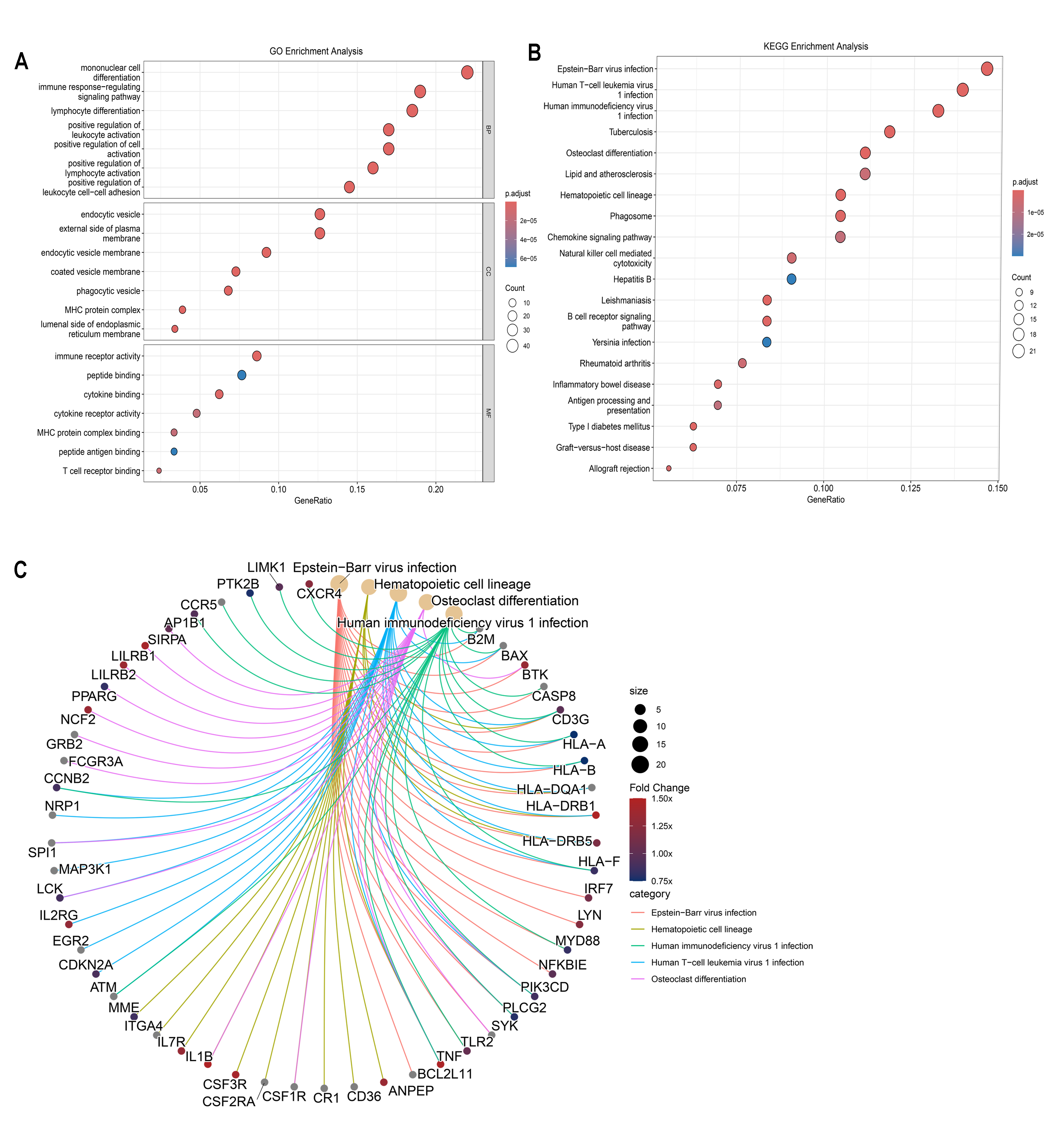

Supplement: Supplementary Figure 1 — Functional analysis of cancer-related genes (CRGs) in AS. (A) GO analysis. (B, C) KEGG analysis. [file Image1.tif]

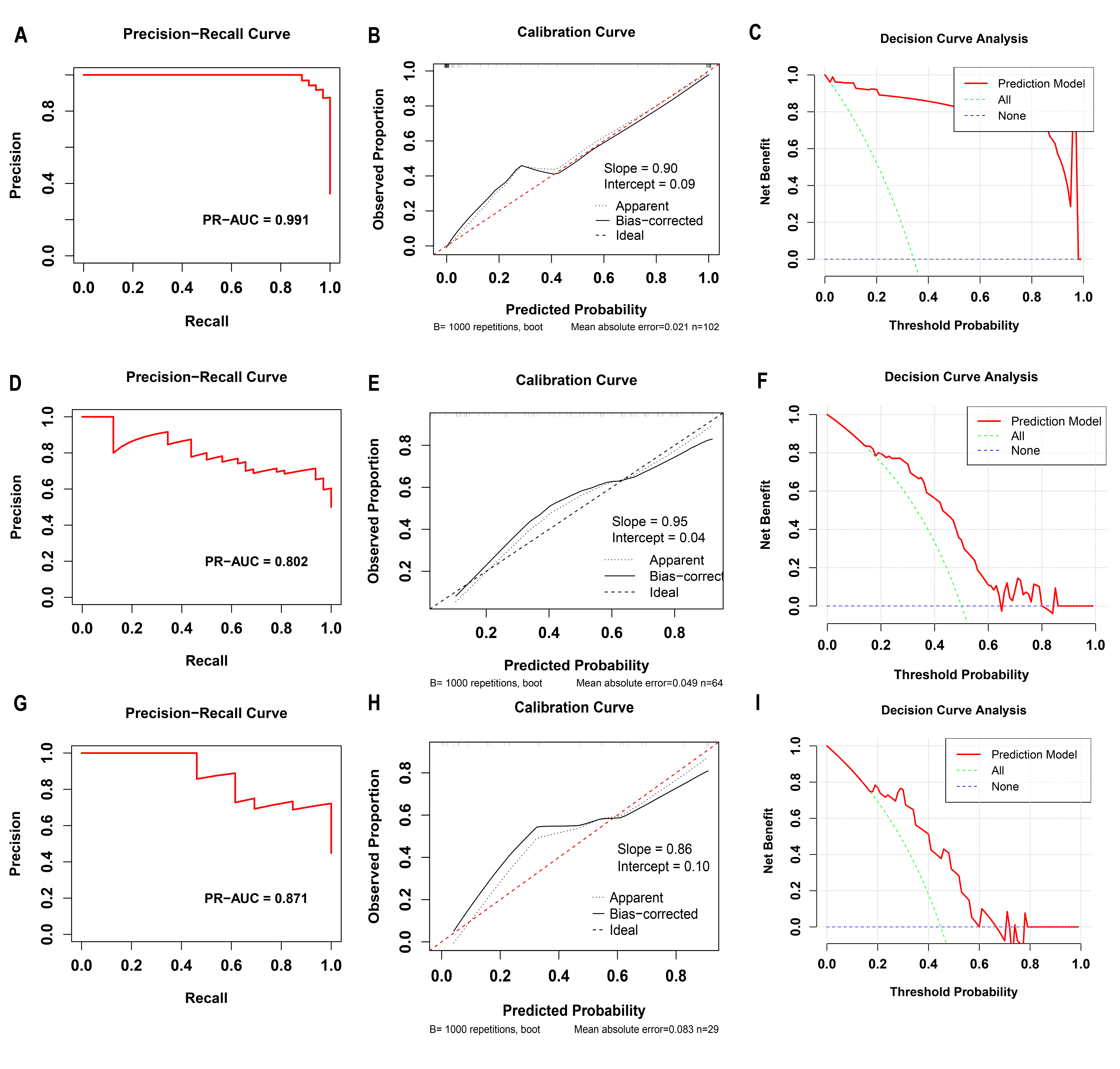

Supplement: Supplementary Figure 2 — Performance evaluation of the machine learning-based atherosclerosis diagnostic prediction model across independent validation datasets. (A–C) Results from the training dataset GSE100927: (A) Precision-Recall curve (PR-AUC = 0.991). (B) Calibration curve (slope = 0.90, intercept = 0.09). (C) Decision curve analysis comparing the model with “All” and “None” strategies. (D–F) Validation results from dataset GSE43292: (D) Precision-Recall curve (PR-AUC = 0.802). (E) Calibration curve (slope = 0.95, intercept = 0.04). (F) Decision curve analysis. (G–I) Validation results from dataset GSE28829: (G) Precision-Recall curve (PR-AUC = 0.871). (H) Calibration curve (slope = 0.86, intercept = 0.10). I Decision curve analysis. All calibration curves were generated using bootstrapping with 1000 repetitions. [file Image2.tif]

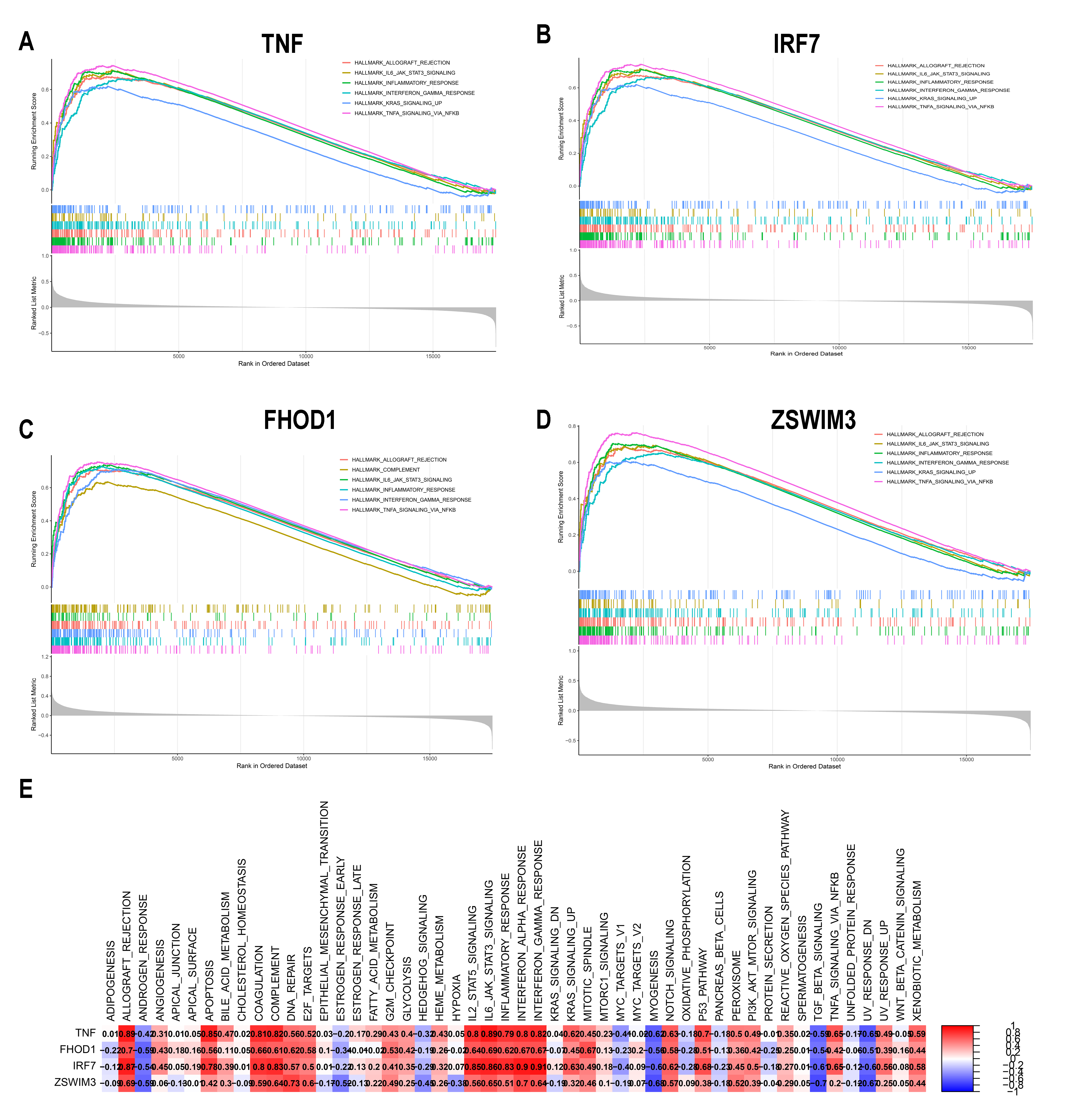

Supplement: Supplementary Figure 3 — Relationship between hallmark pathways and hub genes. (A-D) GSEA analysis of the hub genes, highlighting the top 6 enriched pathways for (A) TNF, (B) IRF7, (C) FHOD1, and (D) ZSWIM3. (E) Correlation analysis between the hub genes and hallmark pathways. [file Image3.tif]

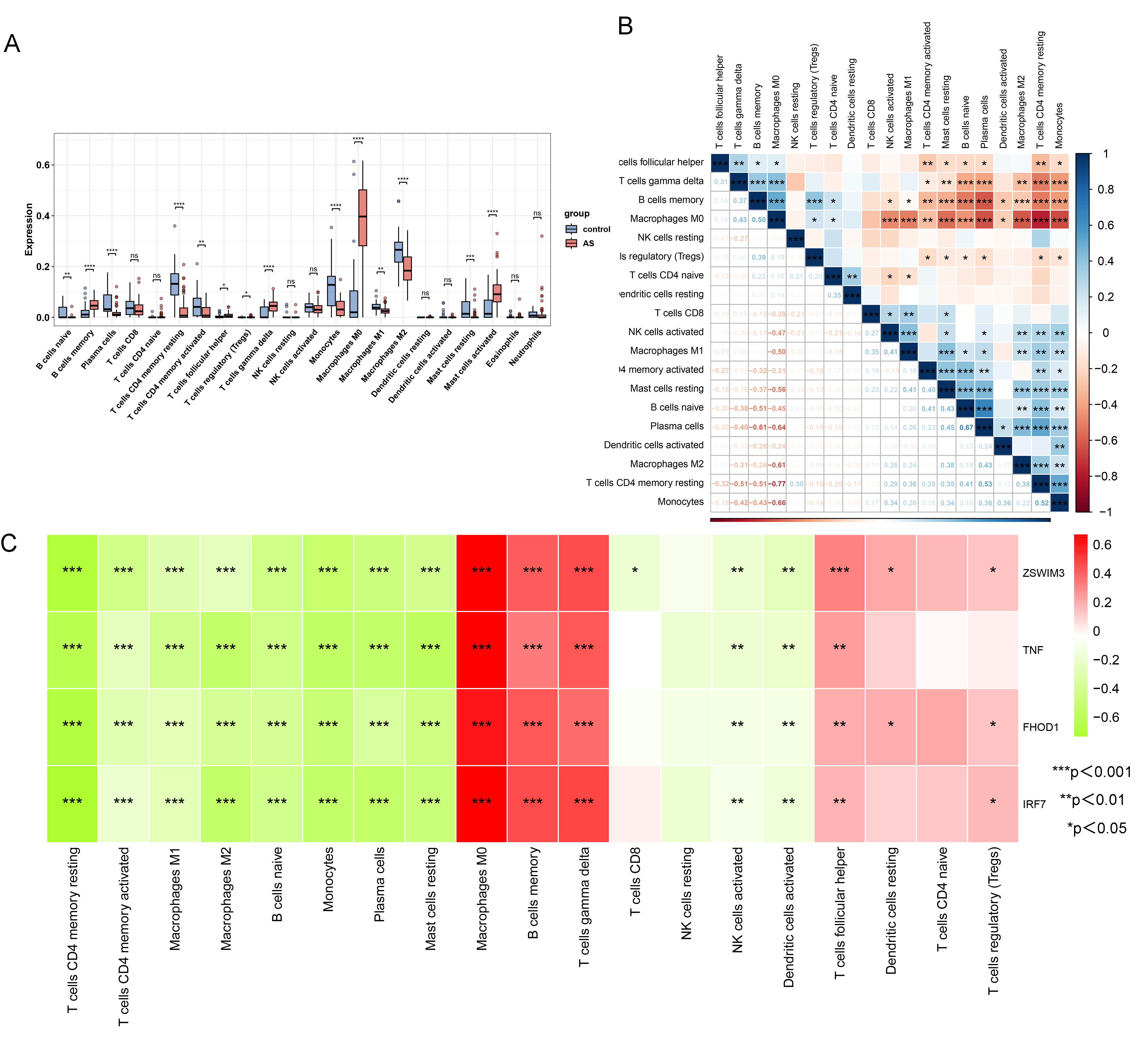

Supplement: Supplementary Figure 4 — Comparison of immune cell type infiltration between AS and controls (GSE100927 dataset) assessed by CIBERSORT. (A) Boxplot illustrating the proportions of immune cells. (B) Correlation matrix of immune cell proportions. *p < 0.05, **p < 0.01, ***p < 0.001. (C) Correlation analysis between hub genes and immune cells. [file Image4.tif]

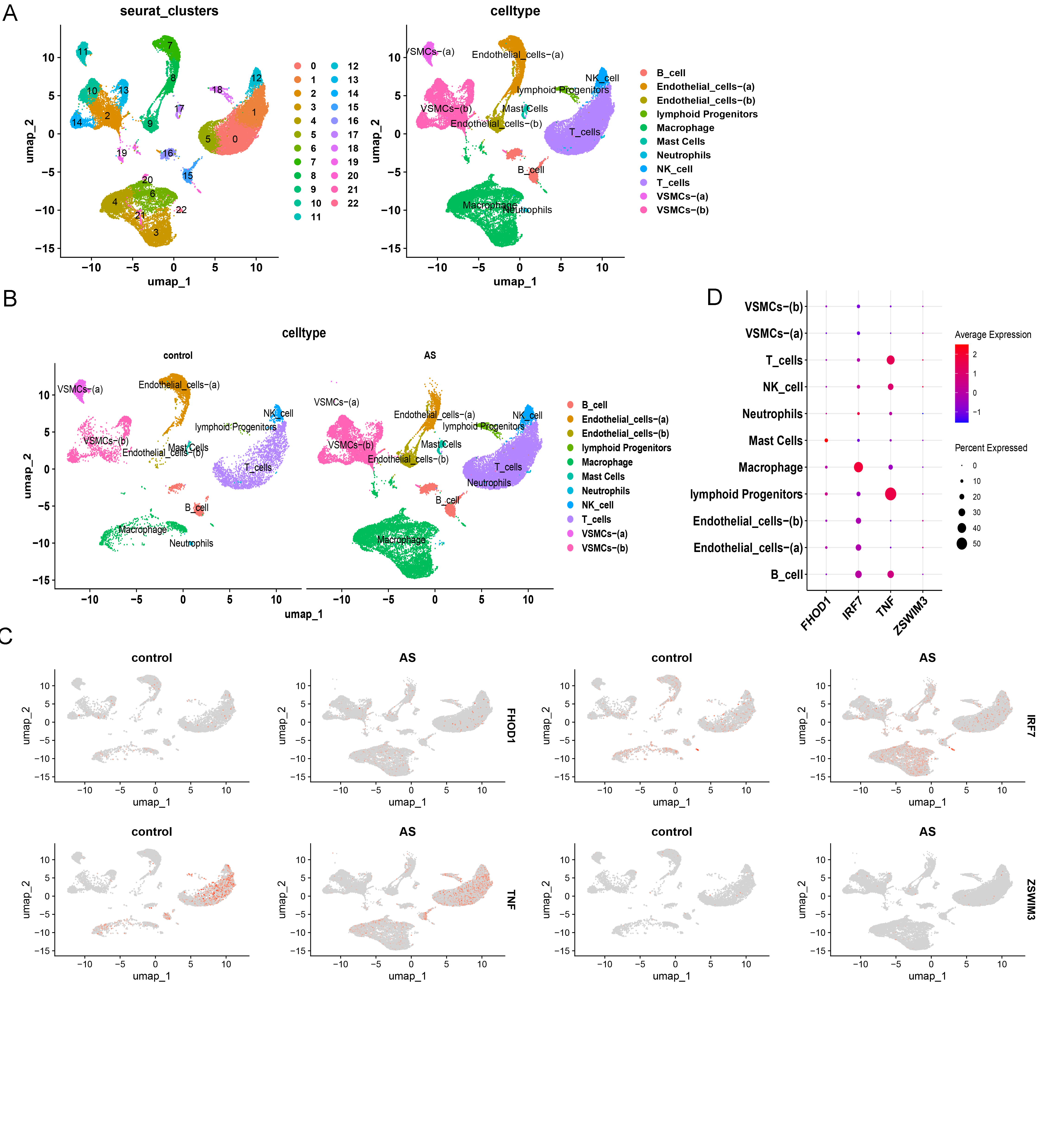

Supplement: Supplementary Figure 5 — Single-Cell analysis after normalization in the GSE159677 dataset. (A) Unified manifold approximation and projection clustering into 22 clusters, and Cells were annotated using CellMarker and singleR. (B) UMAP Visualization: The plot distinctly represents cellular populations in both AS and controls. (C) Feature Plots showing the expression pattern of FHOD1, IRF7, TNF, and ZSWIM3 in calcified atherosclerotic plaque from AS and control groups. (D) Dot plot shows the expression levels of hub genes in each cell cluster. [file Image5.tif]
